# Repetitive Diving in Trained Rats Still Increases Fos Production in Brainstem Neurons after Bilateral Sectioning of the Anterior Ethmoidal Nerve

**DOI:** 10.3389/fphys.2016.00148

**Published:** 2016-04-21

**Authors:** Paul F. McCulloch, Erik A. Warren, Karyn M. DiNovo

**Affiliations:** Department of Physiology, Chicago College of Osteopathic Medicine, Midwestern UniversityDowners Grove, IL, USA

**Keywords:** autonomic reflex, diving response, anterior ethmoidal nerve, c-fos expression, brainstem activation

## Abstract

This research was designed to investigate the role of the anterior ethmoidal nerve (AEN) during repetitive trained diving in rats, with specific attention to activation of afferent and efferent brainstem nuclei that are part of this reflexive response. The AEN innervates the nose and nasal passages and is thought to be an important component of the afferent limb of the diving response. Male Sprague-Dawley rats (*N* = 24) were trained to swim and dive through a 5 m underwater maze. Some rats (*N* = 12) had bilateral sectioning of the AEN, others a Sham surgery (*N* = 12). Twelve rats (6 AEN cut and 6 Sham) had 24 post-surgical dive trials over 2 h to activate brainstem neurons to produce Fos, a neuronal activation marker. Remaining rats were non-diving controls. Diving animals had significantly more Fos-positive neurons than non-diving animals in the caudal pressor area, ventral medullary dorsal horn, ventral paratrigeminal nucleus, nucleus tractus solitarius, rostral ventrolateral medulla, Raphe nuclei, A5, Locus Coeruleus, and Kölliker-Fuse area. There were no significant differences in brainstem Fos labeling in rats diving with and without intact AENs. Thus, the AENs are not required for initiation of the diving response. Other nerve(s) that innervate the nose and nasal passages, and/or suprabulbar activation of brainstem neurons, may be responsible for the pattern of neuronal activation observed during repetitive trained diving in rats. These results help define the central neuronal circuitry of the mammalian diving response.

## Introduction

A reflexive response to diving in mammals is triggered when, upon submersion, the nerves innervating the nose and nasal passages are stimulated with water (Butler and Jones, 1997; McCulloch, 2012; Panneton, 2013). This nasal stimulation initiates significant cardiorespiratory changes that inhibit basic homeostatic reflexes, such as baroreceptor and chemoreceptor reflexes. Apnea accompanied by glottal closure (Dutschmann and Paton, 2002) is a necessity for air-breathing animals to survive underwater. The two other hallmark aspects of the diving response triad, bradycardia and an increase in sympathetic vasomotor tone, constitute an oxygen conserving mechanism that can extend underwater duration (Butler and Jones, 1997; Panneton, 2013).

The anterior ethmoidal nerve (AEN) is a branch of the ophthalmic division of the trigeminal nerve that innervates the external nares and nasal passages (Greene, 1963). As shown in rats, this nerve projects centrally to the ventral medullary dorsal horn (MDH) and adjacent ventral paratrigeminal nuclei of the caudal brainstem (Panneton et al., 2006; Hollandsworth et al., 2009). It is thought that during diving stimulation of the nose and nasal mucosa activates the AEN. The central terminal projections of the AEN then release glutamate (McCulloch et al., 2013) to activate secondary neurons within the ventral MDH and adjacent paratrigeminal nuclei. The activation of these secondary neurons then, in turn, activates other brainstem neurons responsible for the efferent aspects of the diving response. More specifically, neurons in the caudal pressor area (CPA), nucleus of the solitary tract (NTS), raphe nuclei (Ra), rostrolventrolateral medulla (RVLM), catecholaminergic regions (A1, A2, A5, C1, and C2), locus coeruleus (LC), Kölliker-Fuse area (KF), and Parabrachial nucleus (PB) all show an increase in Fos expression during repetitive diving in rats trained to voluntarily swim underwater (McCulloch and Panneton, 2003; Panneton et al., 2010a, 2012a, 2014).

Cardiovascular features of the diving response seen in aquatic and semi-aquatic animals (Butler and Jones, 1997; Panneton, 2013) have been recently documented in conscious rats trained to voluntarily initiate their own dives. For instance, during repetitive trained diving in conscious rats fitted with an implanted arterial blood pressure transmitter, heart rate decreases by approximately 80% and arterial blood pressure increases by 14–42% (McCulloch et al., 2010; Panneton et al., 2010b, 2012a; Chotiyanonta et al., 2013). These responses are qualitatively similar to those seen in anesthetized animals during ammonia stimulation of the nasal passages (Rybka and McCulloch, 2006; Panneton et al., 2010b). Surprisingly rats with bilaterally sectioned AENs are able to initiate a full and complete diving response during repetitive trained diving (Chotiyanonta et al., 2013). The observed diving response in animals with cut AENs is similar to the diving response seen in animals with intact AENs. This finding then challenges the role of the AEN in the reflex initiation of the cardiovascular responses to diving, and leads to an interesting question. Do neurons in the brainstem that show an increase in Fos expression during repetitive diving in intact animals still become activated in the absence of afferent input from the AEN?

To address this question in the present research, we reasoned that the brainstem areas responsible for the efferent aspects of the diving response will still become activated during repetitive diving. We thought this would be true because the cardiovascular responses to repetitive trained diving still persist after bilateral sectioning of the AEN. These areas of the brainstem must become activated to produce the observed cardiorespiratory responses. Thus our first hypothesis was that during repetitive diving in rats with bilaterally sectioned AENs the brainstem areas responsible for the efferent aspects of the diving response will show an increase in neuronal Fos expression compared to non-diving control animals. Our second hypothesis concerned the ventral MDH and paratrigeminal nucleus, the primary recipient zones of the central terminations of the AEN. We reasoned that after bilateral sectioning of the AEN, secondary neurons in this location would receive reduced afferent stimulation from the nose and nasal passages. Thus our second hypothesis was that during repetitive diving in rats with bilaterally sectioned AENs the ventral MDH and paratrigeminal nucleus will not show an increase in neuronal Fos expression compared to non-diving control animals.

## Materials and methods

All procedures for this study were approved by the Midwestern University (Downers Grove) IACUC. Sprague-Dawley rats purchased from a commercial vendor (Harlan) were caged in pairs and housed following the NIH guidelines for the care and usage of laboratory animals. Food and water were available *ad libitum*.

Twenty-four 3 week old rats were trained over 10 weeks to voluntarily swim and then dive through a Plexiglas maze (McCulloch, 2014). The training process began by allowing the rats to gradually learn how to swim through the maze by steadily increasing the swim distances each day. After the rats could successfully swim the full length of the maze, the dive training would begin. The rats would be trained to dive longer distances through the maze each day until they could dive the full 5 m length of the maze in 10–15 s (McCulloch, 2014). To prevent the rats from becoming hypothermic during the training, water temperature was maintained at 30 ± 2°C and rats were dried with a towel between trials.

After all rats mastered the repetitive diving protocol they were then randomly divided into two groups: 12 rats received bilateral sectioning of the AEN, while 12 rats received Sham AEN surgery. Rats were anesthetized using ketamine/xylazine (80/10 mg.kg^−1^ i.p.) and then placed in a stereotaxic device (Kopf Instruments). Under aseptic conditions and using a stereoscopic surgical microscope, the orbit was exposed and the eyeball was retracted laterally. The AEN was identified as it traversed the orbit to pass through the anterior ethmoidal foramen. The AEN was separated from the accompanying artery and vein. In all animals receiving AEN sectioning, a 1 mm piece of the AEN was excised and produced for inspection to confirm the denervation. For Sham surgeries the AEN was isolated but left intact. Surgical procedures were repeated contralaterally. Ketoprofen (5 mg.kg^−1^ s.c.) was given as a post-surgical analgesic both immediately after surgery and 24 h later.

Based on veterinary advice, 9 days of post-surgical recovery elapsed before repetitive dive trials were conducted. Of the 12 rats with bilaterally cut AENs, 6 rats were non-diving controls (Cut-No Dive; C-ND) and 6 rats dived repetitively (Cut-Dive; C-D). Of the 12 rats with Sham AEN surgeries, 6 rats were non-diving controls (Sham-No Dive; S-ND), and 6 rats dived repetitively (Sham-Dive; S-D). Diving rats (C-D and S-D) completed 24 dives in 2 h, while non-diving rats (C-ND and S-ND) remained in their cages.

After completion of the repetitive diving, rats were placed back into their cages for 1 h to allow Fos production within brainstem neurons. The rats were then euthanized with a concentrated pentobarbital solution (Euthasol) and perfused transcardially with saline and then 4% paraformaldehyde. The brain and rostral spinal cord were removed and placed overnight in a 30% sucrose cyropreservative solution. The brains were blocked and cut into 50 μm sections from the rostral spinal cord to the inferior colliculi using a freezing microtome. Next, a 1 in 3 series of brain tissue was subject to immunohistochemistry using goat anti-Fos primary antibody (Santa Cruz, SC-52-G) followed by a biotinylated rabbit anti-goat secondary antibody (Vector, BA5000). The aviden/biotin/perioxidase (ABC) procedure (Vector, PK6100) was applied to enhance immunohistochemical sensitivity. Finally the chromagen DAB + Ni (Vector, SK4100) was applied to the tissue to aid in the visualization of the Fos protein. This immunhistochemical procedure produced a black nucleus in Fos positive neurons. To minimize a possible source of error during tissue processing, each immunohistological series contained 1 animal from each of the 4 groups. Individual brain sections were arranged into serial order, mounted on slides, counter-stained using Neutral Red, and coverslipped using Permount. All slides were re-identified with a random code to blind the group identity of the rat during counting of Fos-positive neurons. This blinding code was not broken until all neuronal counting had been completed.

All sections, from the pyramidal decussation caudally to the inferior colliculus rostrally, were viewed with a Nikon E600 light microscope and compared with a rat brain atlas (Paxinos and Watson, 1998). Fos-positive neurons were counted in several brainstem locations with the aid of Northern Eclipse software (Empix Imaging). In each animal, Fos counts were made bilaterally. A cell containing a sharply defined black staining nucleus was determined to have Fos-like immunoreactivity. Deciding what was, and what was not, a Fos-positive neuron was often difficult. Fos-positive neurons were those neurons that were noticeably darker in intensity compared to other nearby neurons. Consistency in making this determination between brainstem sections in one animal, and between all 24 animals, was the goal. An individual brainstem nucleus was counted by only one researcher in all 24 animals.

**Figure 1:**
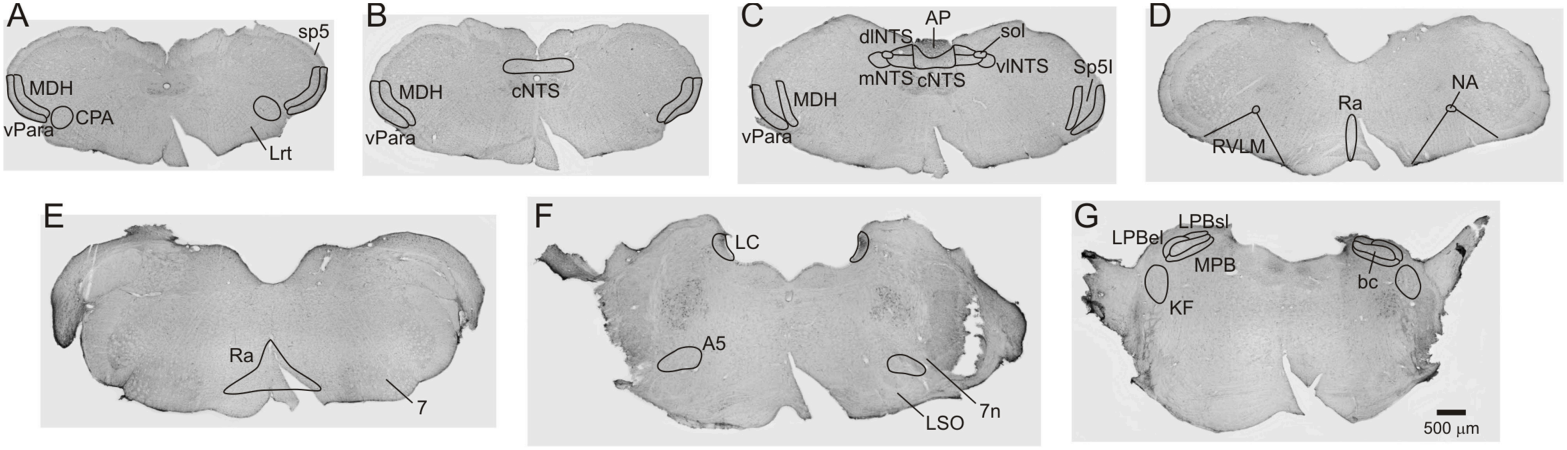
**Brightfield Nissel stained photomicrographs (A-G) from a sham operated non-diving control rat (S-ND) showing extent of brainstem regions inspected for Fos in all animals**. Scale bar in G applies to all panels.

The caudal pressor area (CPA) was found at the level of and immediately adjacent to the caudal pole of the lateral reticular nucleus (6–7 sections). The medullary dorsal horn (MDH) was located along the superficial (laminae I and II) portion of the ventral MDH (9–10 sections). The ventral paratrigeminal nucleus was defined as scattered interstitial neurons located within the ventral half of the spinal trigeminal tract, near the adjacent ventral pole of the MDH (9–10 sections). The ventral MDH and paratrigeminal nucleus extended from the rostral disappearance of the pyramidal decussation caudally to the appearance of Spinal Trigeminal Nucleus Interpolaris near the obex rostrally. The nucleus tractus solitarius (NTS) was divided into commissural (cNTS), dorsolateral (dlNTS), medial (mNTS), and ventrolateral (vlNTS) subregions. Only the cNTS was present caudal to the area postrema (3 sections). When the area postrema was visible (5–7 sections), the cNTS was located immediately ventral to the area postrema; the dlNTS was located bilaterally between the cNTS and solitary tract along the dorsolateral edge of the NTS; the mNTS was located bilaterally immediately ventral to the dlNTS; and the vlNTS was located bilaterally ventral and lateral to the solitary tract. The rostral ventrolateral medulla (RVLM) was defined as being 0–600 μm caudal to the caudal pole of the facial nucleus and within the triangular area that is dorsal to the ventral medullary surface, lateral to a straight line joining the lateral edge of the pyramidal tract to the compact formation of the nucleus ambiguus, and medial to a straight line joining compact formation of the nucleus ambiguus to the lower edge of the trigeminal tract (10–12 sections). The expanded Raphe region (including the Raphe Pallidus, Raphe Magnus, and Raphe Obscurus) was defined as extending caudally from 1200 μm caudal to the caudal pole of the facial nucleus to the exit of the facial nerve rostrally (15 sections). The Raphe region was located along the midline, dorsal to the pyramidal tracts. Rostral to the inferior olivary complex the region expanded laterally to include Raphe Magnus. The A5 region was defined as being immediately dorsal and lateral to the facial nucleus when the 7th nerve or 7th nerve root was visible (5–6 sections). The Locus Coeruleus (LC) was located from the genu of the 7th nerve caudally to the rostral end of the 5th motor nucleus rostrally (6–7 sections). The Parabrachial Nucleus (PB) was found adjacent to the branchium conjunctivum (bc; 5–6 sections). The medial PB (MPB) was immediately medial and ventral to bc, while the lateral PB immediately superior and lateral to bc was divided into external lateral (LPBel) and superior lateral (LPBsl) subregions. The Kölliker Fuse nucleus (KF) was ventral and lateral to the PB (3 sections). See Figure 1 for an outline of anatomical regions.

After all Fos-positive neuronal counts were completed, Two-way ANOVAs (SigmaStat) determined whether the main effects of underwater submergence (no dive; dive) and AEN status (sham surgery; cut) had an effect on the numbers of Fos-positive neurons in each brain region. The level of significance was set at *p* < 0.05. If significant *P*-values were found, Holm-Sidak Pairwise Comparisons were conducted to determine which group differed from the others.

## Results

### Behaviors of diving rats

Of the 12 diving rats, 10 completed 24 diving trials within 2 h; most rats initiated their dives voluntarily within 30 s of being placed in the maze starting area. There were no noted differences between the S-D and C-D rats in the time to initiate their voluntary dives, or in the behavior of the rats while diving. The remaining two rats only completed 18 (a C-D rat) and 22 (an S-D rat) dives within the 2 h. These two rats became increasing reluctant to initiate their dives, sometimes taking 3–4 min after having been placed in the starting area to voluntarily submerge. The Fos labeling from these two rats (see below) was neither quantitatively nor qualitatively different from the remaining 10 diving rats. The average underwater dive duration to complete the maze was 14.0 s for S-D rats, and 13.8 s for C-D rats.

### Fos labeling

**Figure 2:**
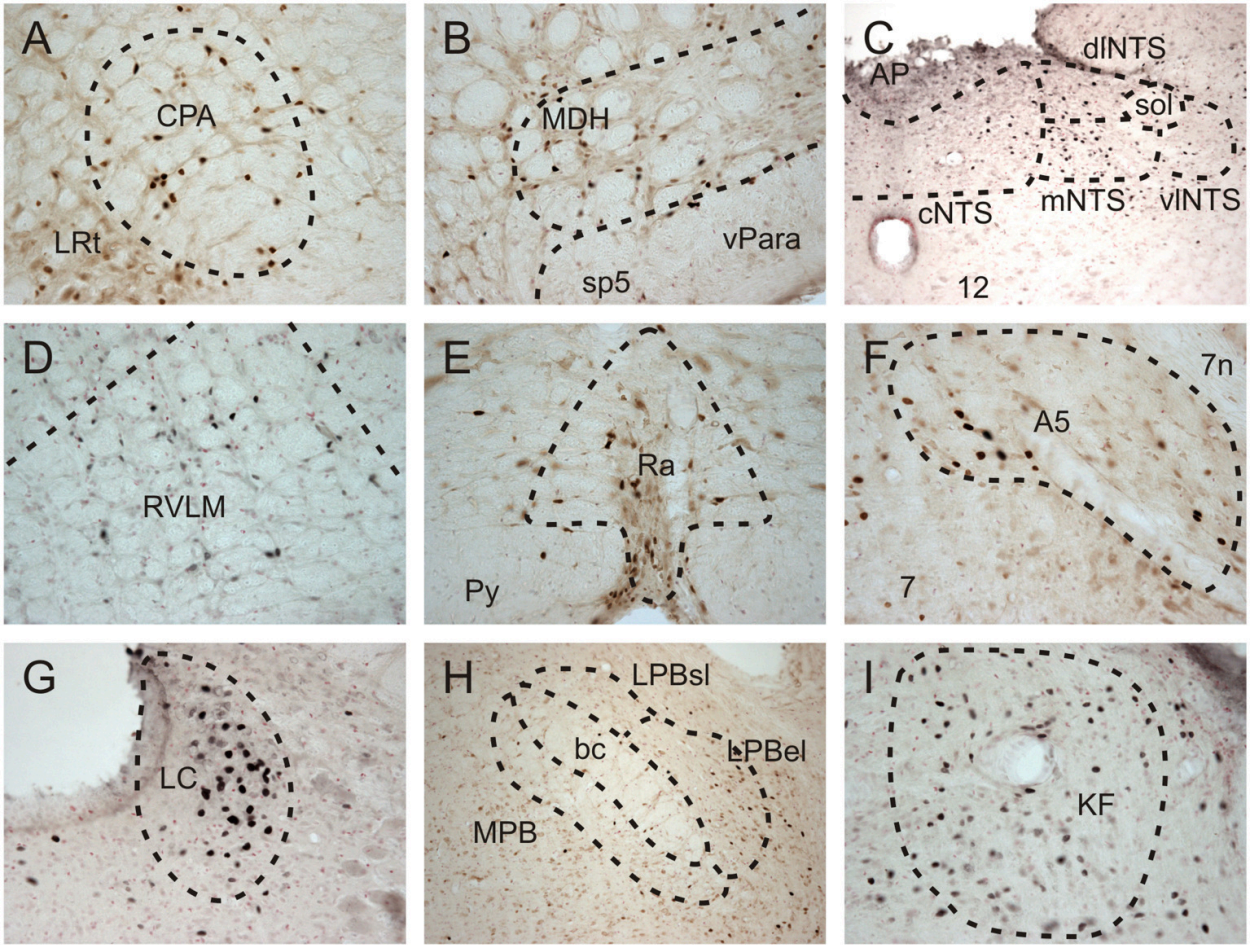
**Brightfield photomicrographs showing Fos-positive neurons in selected brainstem nuclei of repetitively diving rats in which the AEN had been bilaterally sectioned**. Fos-positive neurons were present in the **(A)** caudal pressor area (CPA), **(D)** rostral ventrolateral medulla (RVLM), **(E)** Raphe nuclei (Ra), **(F)** A5 noradrenergic region, **(G)** Locus Coeruleus (LC), and **(I)** Kölliker Fuse region (KF). **(B)** Fos-positive neurons were present in the ventral tip of the superficial laminae of the medullary dorsal horn (MDH), as well as in the ventral paratrigeminal nuclei located within the spinal trigeminal tract (sp5). **(C)** Within the nucleus tractus solitarius at a level just caudal to the obex, Fos-positive neurons were present in the commissural (cNTS), medial (mNTS), and dorsolateral subnuclei (dlNTS), and to a lesser extent within the ventrolateral subnuclei (vlNTS). Within the parabrachial region **(H)**, some Fos-positive neurons were found within the medial parabrachial (MPB) and external lateral subregion of the parabrachial subnuclus (LPB el), while very few Fos-positive neurons were found within the superior lateral subregion of the lateral parabrachial subnuclei (LPBsl). **(B,H)** at 10X; all other panels at 20X.

**Figure 3:**
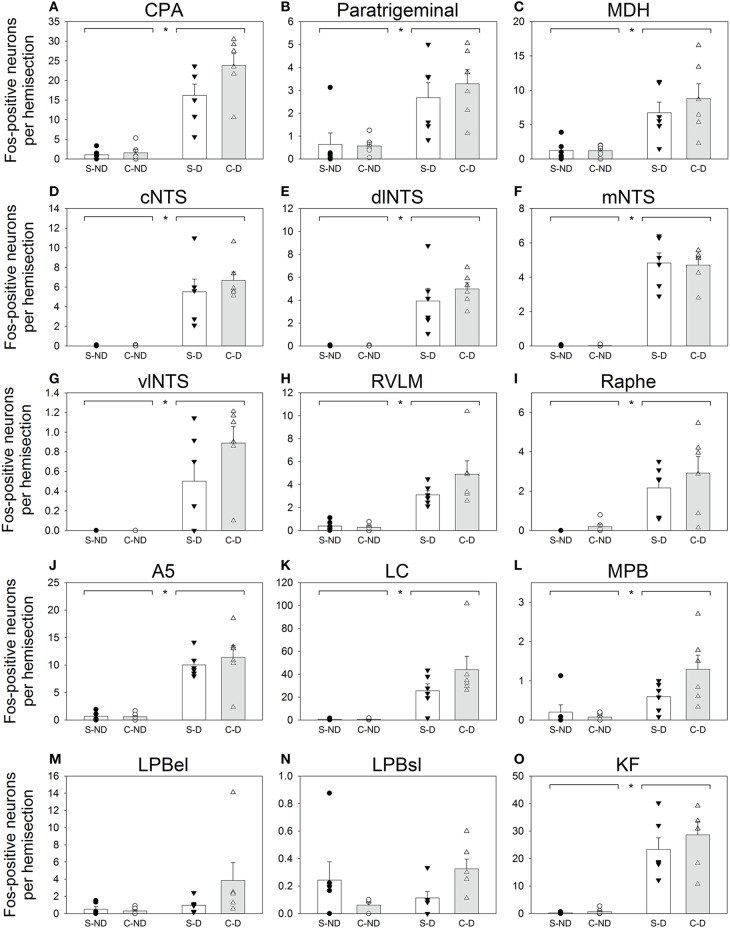
**Counts of Fos-positive neurons for 15 selected brainstem regions (A–O)**. Open bars (± SE) represent rats with bilateral Sham sectioning of the AEN (Sham; S), while shaded bars (± SE) represent rats with bilaterally cut AENs (Cut; C). Two left columns are from rats that did not dive (ND; S-ND and C-ND respectively) and the two right columns are from rats that repetitively dived underwater (D; S-D and C-D respectively). Symbols (circles and triangles) represent Fos counts from individual animals within each of the 4 groups. Horizontal bars separated by asterisks (^*^) along the top edge of the panels represent significant differences between the non-diving (S-ND and C-ND) and diving groups (S-D and C-D) as indicated by Two way ANOVAs.

Fos-positive neurons were visualized throughout the brainstem of all diving and non-diving rats (Figure 2). The 15 nuclei/subregions chosen for Fos quantification in the present study were previously identified as showing an increase in activity (i.e., an increase in Fos expression) during repetitive trained diving in rats (McCulloch, 2005; Panneton et al., 2010a, 2012a). Other brainstem areas occasionally contained sporadic Fos labeling, but were not quantified. Figure 3 includes both the mean and SEs of the Fos counts (bars) and the Fos counts from individual animals (symbols), as sometimes an individual animal within a group had a Fos count differ from others in that group by close to an order of magnitude (i.e., see Figures 3K,M,N).

Some brainstem areas of the diving rats, notably CPA, A5, LC, and KF, were densely labeled with Fos-positive neurons (Table 1 and Figures 2, 3). The NTS of non-diving rats had no Fos-labeling, while the cNTS, dlNTS, and mNTS subregions of diving rats had approximately 5 Fos-positive neurons each. Other brainstem regions with minimal Fos labeling in the non-diving rats included CPA, ventral paratrigeminal, RVLM, Ra, LC, and KF. All PB subregions generally had minimal Fos-labeling, even in the diving rats. Ventral MDH and paratrigeminal regions had some Fos-positive neurons in the non-diving rats, but showed a 6–7-fold increase in Fos labeling in the diving rats.

**Table 1:** **Number of Fos-positive neurons in each designated brainstem area**.

**Brainstem area**	**S-ND**	**C-ND**	**S-D**	**C-D**	**ND vs. D (*P*-value)**
**CPA**	1.1±0.5	1.6±0.8	16.2±2.9	23.8±3.0	< 0.001
**Ventral Trigeminal**					
Paratrigeminal	0.6±0.5	0.6±0.2	2.7±0.7	3.3±0.6	< 0.001
Superficial MDH	1.2±0.6	1.2±0.3	6.7±1.6	8.8±2.2	< 0.001
**NTS**					
cNTS	0.0±0.0	0.0±0.0	5.5±1.3	6.7±0.9	< 0.001
dlNTS	0.0±0.0	0.0±0.0	3.9±1.1	5.0±0.6	< 0.001
medNTS	0.0±0.0	0.0±0.0	4.8±0.6	4.7±0.4	< 0.001
vlNTS	0.0±0.0	0.0±0.0	0.5±0.2	0.9±0.2	< 0.001
**RVLM**	0.4±0.2	0.3±0.1	3.1±0.3	4.9±1.2	< 0.001
**Raphe**	0.0±0.0	0.2±0.1	2.2±0.5	2.9±0.8	< 0.001
**A5**	0.7±0.4	0.6±0.3	10.0±0.9	11.4±2.2	< 0.001
**LC**	0.6±0.2	0.5±0.3	25.6±6.0	43.9±11.7	< 0.001
**Parabrachial**					
MPB	0.2±0.2	0.1±0.0	0.6±0.1	1.3±0.4	0.001
LPBel	0.5±0.3	0.3±0.2	1.0±0.3	3.9±2.1	0.073
LPBsl	0.2±0.1	0.1±0.0	0.1±0.0	0.3±0.1	0.404
KF	0.3±0.1	0.7±0.4	23.3±4.3	28.7±4.8	< 0.001

Counts are expressed as number of neurons (± standard error) per hemisection. S-ND is Sham, No Dive (N = 6); C-ND is AEN cut, No Dive (N = 6); S-D is Sham, Dive (N = 6); C-D is AEN Cut, Dive (N = 6). Two way ANOVAs indicated that the main effect of underwater submergence (no-dive; dive) produced a significant increase in the diving rats compared with the non-diving rats in all brain regions except LPBe and LPBs. There was no main effect of AEN status (sham; cut), nor was there any interaction effect (underwater submergence X AEN status), for any brainstem area.

For most brainstem areas counted (see Table 1 and Figure 3), the diving rats (S-D and C-D) had significantly more Fos-positive neurons than the non-diving rats (S-ND and C-ND) as determined by Two-way ANOVAs. The only subregions where this was not found were LPBel and LPBsl. There was no main effect of AEN status, nor was there any interaction effect (underwater submergence X AEN status), for all 15 brainstem nuclei. A general trend was that C-D rats had more Fos-positive neurons than S-D rats.

## Discussion

Results from the present experiments indicate specific brainstem nuclei are activated during voluntarily-initiated repetitive trained diving in rats. This determination was made by counting Fos-positive neurons in 15 brainstem nuclei/subregions in both diving rats and their non-diving controls. The increases in Fos-positive neurons observed during diving were not significantly different in rats with and without intact AENs. This calls into question the necessity of the AEN providing the afferent information required to initiate the diving response. We also found that in rats both with and without intact AENs there is a significant increase in Fos labeling within the ventral MDH and paratrigeminal nuclei during repetitive diving.

### Technical considerations

The counting of Fos-positive neurons is a useful technique to elucidate the afferent and efferent limbs of a reflex pathway (McCulloch and Panneton, 1997). However the technique is not without theoretical limitations (Dragunow and Faull, 1989; Kovacs, 2008; Guyenet, 2014). Immunohistological procedures are another limitation that can cause variations in staining intensity, making difficult the determination of whether or not a moderately stained neuron is considered Fos-positive. To minimize this confounding variable, only one of us counted any particular brainstem nucleus/subregion, and we strived for consistency in determining a Fos-positive neuron both within and between animals. The blinding of individual slides when counting Fos-positive neurons helped minimize researcher bias. Additionally, even when such staining considerations were accounted for, the number of Fos-positive neurons within a particular brainstem nuclei can just vary widely between animals, even when all animals are from a single group (see Figure 3). Altogether, these considerations can complicate quantitative comparisons made between different Fos studies.

The experimental design of the experiments did not include comparison between swimming and diving animals because it has previously been shown that the diving response is not initiated when rats just swim on the surface of the water (McCulloch et al., 2010; Panneton et al., 2010b). In most brainstem regions, the number of Fos positive neurons is significantly greater in diving rats compared with swimming rats (Panneton et al., 2012a). Additionally, Fos production in brainstem nuclei are generally not different between Swimming rats and Control rats remaining in their cage, and in many cases Fos counts are lower in the Swimming rats compared with Control rats (Panneton et al., 2012a). Accordingly, the decision to not include swimming animals in the experimental design was made, which decreased the number of animals used in these experiments.

### Activated brainstem nuclei

In the present study there was a significant increase in the number of Fos-positive CPA neurons in diving rats compared with non-diving rats. A similar increase in CPA activation was also seen in voluntarily trained diving rats by Panneton et al. (2012a). This suggests that the CPA could possibly be part of the neuronal circuitry of the diving response. The CPA is involved in cardiovascular regulation as glutamate or DLH microinjections into this area cause a significant increase in arterial blood pressure (Gordon and McCann, 1988; Natarajan and Morrison, 2000; Sun and Panneton, 2002). However, inhibition of the CPA with glycine or muscimol does not alter the cardiorespiratory responses produced by nasal stimulation in anesthetized animals (Panneton et al., 2008). Thus the actual role of the CPA during conscious voluntarily diving in rats requires further investigation. Additionally, although the A1 noradrenergic area and the CPA are closely located anatomically, these 2 cell groups appear to cause increases in arterial pressure through different mechanistic pathways (Sun and Panneton, 2002).

The ventral portion of the superficial laminae of the MDH receives ipsilateral central terminations of nerves that innervate the nose and nasal passages (Anton and Peppel, 1991; Panneton, 1991; Panneton et al., 2006; Hollandsworth et al., 2009). The nearby paratrigeminal nucleus consists of an interstitial system of displaced neuropil extending both dorsally and ventrally within the spinal trigeminal tract (Chan-Palay, 1978; Phelan and Falls, 1989). The ventral paratrigeminal nucleus receives somatosensory afferents from trigeminal nerves innervating the nose, cornea, and anterior facial skin (Shigenaga et al., 1986; Panneton, 1991; Hollandsworth et al., 2009). In contrast, the dorsal paratrigeminal nucleus receives trigeminal, glossopharyngeal and vagal inputs (Shigenaga et al., 1986; Panneton, 1991; Saxon and Hopkins, 2006) and is involved in baroreceptor reflexes and cardiovascular regulation (De Sousa Buck et al., 2001; Yu and Lindsey, 2003; Junior et al., 2004). Neurons within the ventral MDH and paratrigeminal nucleus show an increase in the number of Fos-positive neurons, and thus are activated, both during repetitive trained diving in conscious animals (present study; McCulloch, 2005; Panneton et al., 2012a) and nasal stimulation in anesthetized animals (Dutschmann and Herbert, 1997; McCulloch and Panneton, 1997; Rybka and McCulloch, 2006). After activation of the MDH neurons following nasal stimulation, presumably a signal is passed to other brainstem neurons important in both the integrative and efferent aspects of the diving response (Panneton et al., 2000, 2006). Therefore secondary neurons within the ventral MDH and paratrigeminal nucleus constitute an important afferent aspect of the neuronal circuitry of the diving response. These nuclei provide the link between the sensory stimulation of the nasal passages and the physiological efferent reflex responses. For instance, there is a polysynaptic excitatory glutamatergic pathway from the trigeminal nerve to cardiac vagal neurons (Gorini et al., 2009) that presumably is the source of the intense bradycardia seen during diving. But, as Panneton et al. (2012a) commented, it is surprising then that although there is a significant increase in the number of Fos-positive neurons within the ventral MDH during trained repetitive diving, the absolute number of Fos-positive MDH neurons is relatively low considering their important function in the initiation of this autonomic reflex.

The NTS is a primary relay for visceral afferents and a principal integrative center for circulatory control (Potts, 2002; Andresen et al., 2004; Guyenet, 2006). In all 4 NTS subregions inspected (cNTS, dlNTS, mNTS, and vlNTS), there was a significant increase in the number of Fos-positive neurons during repetitive trained diving. Increases in Fos labeling within the NTS during diving in rats have been previously reported (Panneton et al., 2012a). Although the absolute numbers of Fos-positive neurons in these areas were not excessive during diving, all four areas showed virtually no Fos labeling in the non-diving control animals. Clearly the NTS is activated during diving and so could possibly constitute part of the reflex circuitry of the diving response. Alternatively the increase in NTS Fos labeling during diving perhaps could be due to an increase in locomotory activity in general (Potts, 2001). This however seems unlikely as surface swimming without submersion does not increase NTS Fos labeling (Panneton et al., 2012a). Caudal to obex cNTS contains noradrenergic A2 neurons (Rinaman, 2011), and 53% of A2 neurons are activated during voluntary diving (McCulloch and Panneton, 2003). The cNTS receives afferent fibers from arterial chemoreceptors monitoring blood gases (Guyenet, 2014), and so developing hypoxia may have caused activation of the cNTS (Guyenet, 2006). However, the short underwater duration (14 s) is well within the aerobic diving capabilities of rats (Panneton et al., 2010a). It is therefore unlikely that our diving rats were experiencing an oxygen deficit while underwater. The dlNTS and mNTS receive afferent fibers from carotid and aortic baroreceptors (Andresen and Kunze, 1994; Ciriello et al., 1994). Since mean arterial pressure significantly increases in rats while diving voluntarily (Panneton et al., 2012a; Chotiyanonta et al., 2013), the activation of the dlNTS neurons may be secondary to the arterial pressure changes and not part of the diving response circuitry itself. mNTS neurons are likely involved in interoceptive visceral functioning, and contains both A2 noradrenergic neurons and C2 adrenergic neurons (Rinaman, 2011). However, only 17% of tyrosine hydroxylase positive neurons at the level of AP are activated during voluntary diving (McCulloch and Panneton, 2003). It is unknown whether mNTS neurons activated in the present study were adrenergic or noradrenergic, or what the specific function of the activated mNTS neurons may be. The vlNTS receive respiratory afferents (Andresen and Kunze, 1994), and may promote the transition from inspiration to expiration (Wasserman et al., 2000). This transition could be important for the necessary apnea that occurs during underwater submersion.

The RVLM is a heterogeneous region regulating both the hypothalamic pituitary axis and autonomic nervous system, especially the control of arterial blood pressure and respiration (Guyenet, 2006, 2014; Guyenet et al., 2013). Barosensitive RVLM neurons projecting to sympathetic preganglionc neurons in the thoracolumbar spinal cord are a principal effector of the baroreflex (Aicher et al., 2000). Yet during nasal stimulation 62% of sympathoexcitatory bulbospinal neurons in the RVLM show an increase in firing rate despite a concomitant increase in arterial blood pressure (McCulloch et al., 1999). Also, nearly 50% of adrenergic C1 neurons contained within the RVLM express Fos during trained diving (McCulloch and Panneton, 2003). In the present study there was a significant increase in Fos-positive neurons in RVLM during trained diving, which was also found by Panneton et al. (2012a). The activity of RVLM barosensitive neurons can determine whether background sympathetic vasomotor tone is enhanced or withdrawn (Guyenet, 2006). Presumably, the activation of RVLM neurons is involved in mediating the peripheral vasoconstriction that contributes to the increase in arterial pressure observed during diving (Panneton et al., 2012a; Chotiyanonta et al., 2013).

The location of central respiratory chemoreceptors are numerous and diffuse (Richerson et al., 2005; Nattie and Li, 2006, 2012; Guyenet, 2014). One such region is the extended Raphe region that includes the midline serotonergic neurons of the Raphe Pallidus, Raphe Magnus, and Raphe Obscurus (Richerson, 2004; Pilowsky, 2014). The extended Raphe neurons are sensors of arterial blood carbon dioxide levels, and they influence breathing, cardiovascular control, and autonomic output (Richerson, 2004; Smith et al., 2013). There was a significant increase in Fos-positive neurons in the extended Raphe during diving, similar to previous results (Panneton et al., 2010a, 2012a). It could be possible the short underwater duration (14 s) of the rats in the present study is long enough to increase the tightly controlled P_a_CO_2_ to levels causing activation of chemosensitive Raphe nuclei during diving. However, even if this is the situation, during wakefulness the primary effect of serotonergic neurons on breathing is excitatory (Richerson, 2004), and during diving there is an obligatory cessation of breathing. Additionally, neurons expressing Fos in animals exposed to hypercapnea are not necessarily central respiratory chemoreceptors, and therefore such Fos data must be interpreted cautiously (Guyenet, 2014). It is also possible Raphe neurons increased their Fos expression during diving due to some other non-respiratory function (Richerson, 2004; Pilowsky, 2014).

Located in the pons, the A5 region is a principal source of noradrenergic input to sympathetic preganglionic neurons (Loewy et al., 1979). A5 neurons are thought to primarily regulate autonomic functioning, including both cardiovascular reflexes and respiratory rhythm generation, as well as nociceptive transmission (Sun, 1995; Hilaire et al., 2004; Kanbar et al., 2011). Nearly 65% of noradrenergic A5 neurons express Fos during trained diving (McCulloch and Panneton, 2003). In the present study there was a significant increase in Fos-positive neurons in A5 during trained diving, which was also found by Panneton et al. (2012a). Activation of A5 neurons may contribute to visceral sympathetic outflow during diving, and, along with the RVLM, may mediate the increased arterial pressures observed during diving (Panneton et al., 2012a; Chotiyanonta et al., 2013).

LC, the noradrenergic A6 region, is an important pontine nucleus helping regulate stress, anxiety, sensory processing, and behavioral orientation (Aston-Jones et al., 1995; Itoi and Sugimoto, 2010). There is a significant and large increase in the absolute numbers of Fos-positive neurons within LC during voluntary diving in rats (present study; McCulloch and Panneton, 2003; Panneton et al., 2012a). However, LC appears not be a necessary component of the diving response circuitry. This is because the cardiorespiratory responses to nasal stimulation persist even after a pontomedullary transection that separates LC from the medulla (Panneton et al., 2012b). Thus it is possible the large increase in LC labeling observed during diving is due to the unique somatosensory experience, or perhaps the “stressfulness,” of underwater submersion rather than being a part of the diving response circuitry *per se*.

PB and KF help modulate autonomic activity, including the blood pressure, breathing rate and amplitude (Guyenet, 2014). In general the Fos labeling within either the medial (MPB) or lateral (LPBel and LPBsl) PB was quite sparse during diving. Additionally, when the AEN was intact, there were no significant differences between the PB of diving rats compared with non-diving rats. Since the LPB contributes to the patterning of inspiratory outflow (Guyenet, 2014), and apnea occurs during diving, it is not surprising the PB region had only scattered Fos labeling in the diving rats. However our findings are in contrast to Panneton et al. (2012a) who found substantially more Fos-positive neurons within the PB region. They also found significant increases in Fos labeling within LPBel and LPBsl in diving rats compared to non-diving control rats. At present there is no explanation for these discrepant findings. In comparison, the large increase in Fos-positive neurons found within KF during diving was also found by Panneton et al. (2012a). KF has previously been found to be important in trigeminal-autonomic reflexes (Dutschmann and Herbert, 1998), including the development of the apnea and bradycardia (Dutschmann and Herbert, 1996). The excitatory drive to post-inspiratory neurons located in the ventral respiratory column by KF neurons (Guyenet, 2014) may facilitate the diving apnea, especially the inspiratory-expiratory phase transition (Dutschmann and Herbert, 2006). However it is possible that neither PB nor KF are a necessary component of the diving response circuitry, because cardiorespiratory responses to nasal stimulation persist after pontomedullary transection (Panneton et al., 2012b).

### Effect of bilateral sectioning of the AEN

A significant new finding of the present research is 13 of the 15 areas of the brainstem inspected showed an increase in Fos expression, and thus presumably were activated, during repetitive trained diving in rats with bilaterally sectioned AENs. There were no differences in Fos expression during repetitive diving between the two groups of diving animals (S-D and C-D). Thus, based on these results, we accept our first hypothesis that brainstem areas responsible for the efferent aspects of the diving response will show an increase in neuronal Fos expression compared to non-diving control animals. In fact the general trend was that the C-D rats had slightly more Fos-positive neurons than the S-D rats. The cardiovascular responses to repetitive trained diving still persist after bilateral sectioning of the AEN (Chotiyanonta et al., 2013). Rats without intact AENs show a 78% decrease in heart rate and a 39% increase in mean arterial pressure during diving, responses virtually identical to those seen in intact animals. It is therefore logical that the identified efferent areas of the brainstem would become activated during diving to produce the observed cardiorespiratory responses.

Our results also showed two areas of the brainstem involved in the afferent aspects of the diving response circuitry, the ventral superficial MDH and ventral paratrigeminal nucleus, had significant increases in Fos expression in rats with bilaterally sectioned AENs compared with their non-diving controls. There were no differences in Fos expression within the ventral MDH and paratrigeminal nucleus during repetitive diving between the two groups of diving animals (S-D and C-D). Thus we were unable to accept our second hypothesis that the MDH will not show an increase in neuronal Fos expression compared to non-diving control animals. This finding is rather interesting. Why did these afferent areas show an increase in Fos labeling in the rats with bilaterally sectioned AENs during diving? Presumably this surgery would have reduced afferent sensory stimulation originating from the nose and nasal passages. This should have reduced, not increased, the activity of the secondary neurons of the ventral MDH and paratrigeminal nucleus during diving. An even more interesting related question is how do the areas of the brainstem responsible for the efferent aspects of the diving response become activated during diving in the absence of AEN input?

There are two possibilities to explain how Fos expression is increased within brainstem nuclei during diving in rats with bilaterally sectioned AENs. (1) Other nerves that innervate the nose and nasal passages, in addition to the AEN, take over the function of the AEN after the AENs are cut bilaterally; (2) There is another way of initiating the diving response that uses a non-reflexive mechanism that involves the cortex.

In addition to the AEN, other nerves, such as the infraorbital, superior alveolar and nasopalatine nerves, also innervate the nose and nasal passages (Greene, 1963). After bilateral sectioning of the AEN, these other nerves could possibly provide the afferent input capable of activating brainstem neurons which then initiates the diving response (Chotiyanonta et al., 2013; Panneton, 2013). Presumably during diving this non-AEN innervation would provide sufficient afferent traffic to activate neurons within the ventral MDH and paratrigeminal nucleus in a fashion similar to when the AENs are intact. However, this possibility would require some sort of functional recovery to occur within the MDH after AEN sectioning. The efferent cardiorespiratory responses to nasal stimulation are severely attenuated in anesthetized rats with bilaterally cut AENs (Rybka and McCulloch, 2006). However, voluntarily diving rats are able to initiate a full and complete diving response after bilateral sectioning of the AENs (Chotiyanonta et al., 2013). The discrepancy between the conclusions found by Rybka and McCulloch (2006) and those by Chotiyanonta et al. (2013) may be due to the timing used in their experiments. Rybka and McCulloch (2006) used anesthetized animals in which the AENs were cut acutely, and found the nasopharyngeal response was attenuated. Chotiyanonta et al. (2013) recorded the diving response in voluntarily diving conscious rats 7 days after their AENs were cut, and found a full and complete cardiorespiratory response to diving. Furthermore, Chotiyanonta et al. (2013) used these same chronically AEN sectioned rats in acute experiments identical to those conducted by Rybka and McCulloch (2006). They found 9 days after AEN denervation surgeries the cardiorespiratory responses to nasal stimulation with ammonia vapors or water in anesthetized animals were identical to those from Sham operated animals. Chotiyanonta et al. (2013) discussed the possibility of neuronal rewiring within the MDH to explain the return of the diving response after AEN sectioning. This rewiring would have to include neuronal plasticity that develops functional connections between non-AEN but nasally-innervating primary afferent fibers and secondary neurons within the MDH. But even if the AEN has a unique afferent input with central projections beyond the trigeminal sensory complex (Panneton and Gan, 2014), there must have been a recovery of function in the animals with bilateral sectioned AENs used by Chotiyanonta et al. (2013), since there was a return of the reflex cardiorespiratory responses to nasal stimulation in these anesthetized animals.

The second possibility to explain how Fos expression is increased within brainstem nuclei during diving in rats with bilaterally sectioned AENs could involve descending cardiorespiratory signals originating from the cortex. For instance, the medial prefrontal cortex and insular cortex participate in specific aspects of central circulatory control, such as baroreflex and conditioned cardiovascular responses (Verberne and Owens, 1998; Shoemaker and Goswami, 2015). Many aquatic animals show an anticipatory bradycardia in advance of submersion, and a return of heart rate to pre-diving values before returning to the water surface (Butler and Jones, 1997; Panneton, 2013). This suggests suprabulbar or cortical influences may modulate the cardiorespiratory reflex initiated by nasal stimulation (Butler and Jones, 1997). In addition, an intensification of the diving bradycardia can develop during increasingly stressful situations in ducks and muskrats (Furilla and Jones, 1986; McCulloch and Jones, 1990), although apparently not in rats (McCulloch et al., 2010). Thus the “type” of dive can affect the observed cardiovascular responses in many diving species (Butler and Jones, 1997). Based on this consideration, we have used the term “repetitive trained diving” in the present research, rather than the term “voluntary diving” that we have used previously (McCulloch et al., 2010; Chotiyanonta et al., 2013; McCulloch, 2014). “Voluntary diving” is often reserved to describe the response seen in animals diving in their natural environment, rather than diving in a tank located in a laboratory (Butler and Jones, 1997). It is possible the cortical modulation of the cardiovascular system that allows many aquatic animals to consciously alter the reflexive aspects of the diving response also exists in rats. This could explain how, in the absence of afferent input from the AEN, the cardiovascular response is still observable (Chotiyanonta et al., 2013) and there are Fos increases in efferent brainstem nuclei (present research) during diving in rats with bilaterally sectioned AENs. A conscious decision to voluntarily submerge underwater could include descending signals that induce brainstem nuclei involved in autonomic control to produce alterations in heart rate and sympathetic tone. Furthermore, there were no overt behavioral differences during diving between the animals with and without intact AENs. This suggests that the reduction in somatosensory innervation from the nasal region did not affect the rats' decision to voluntarily initiate their dives. Although 2 rats did not complete the complete 24 dive trials, one of these 2 animals had intact AENs, and thus bilateral sectioning of the AENs was not the cause of both animals having a reluctance to dive.

It is noteworthy that these 2 alternate possibilities are not mutually exclusive. The diving response could be initiated by both a reflex due to stimulation of the nasal passages and through suprabulbar control. Aquatic animals just may have a better developed capability to alter their autonomic nervous system during diving than do non-aquatic animals. But this does not preclude the possibility that this cortical mechanism is also present in rats, and is only unmasked after the AENs are cut bilaterally. If there is such a descending cortical signal in rats, this would explain how the efferent areas of the brainstem would become activated during diving. However this would not explain how the afferent areas of the circuitry, the ventral MDH and paratrigeminal nucleus, would produce an increase in Fos labeling during repetitive diving. One possibility is a descending cortical signal to the recipient zone of somatosensory sensation, the dorsal horn. There is a direct ascending pathway from the MDH to the anterior cingulate cortex (Iwata et al., 2011), and many areas of the brainstem do receive direct projections from the ventral MDH (Panneton et al., 2006). However there appears to be no evidence of direct descending pathways to the MDH from cortical regions such as the medial prefrontal cortex and insular cortex (Verberne and Owens, 1998). The second possibility is that during diving after bilateral sectioning of the AENs the alternate nasal nerves provided sufficient afferent sensation to produce activation, and thus an increase in Fos labeling, of secondary neurons within the ventral MDH and paratrigeminal nucleus.

## Conclusions

Specific brainstem nuclei are activated during repetitive trained diving in rats compared to control non-diving rats. This was shown by significant increases in the number of Fos-positive neurons within CPA, ventral MDH, ventral paratrigeminal nucleus, NTS, RVLM, Raphe, A5, LC, medial PB, and KF during diving. These results echo those found previously (Panneton et al., 2010a, 2012a). Furthermore, the increases in Fos-positive neurons observed in brainstem areas responsible for both afferent and efferent aspects of the cardiorespiratory responses to diving were not significantly different in rats with and without intact AENs. This suggests that the AEN is not a required feature of the diving response central neuronal circuitry. After bilateral sectioning of the AEN, brainstem areas that are part of the afferent aspect of the diving response, the ventral MDH and paratrigeminal nucleus, could have been activated by other nerves that innervate the nose and nasal passages. For this to occur, neuronal plasticity would be necessary within the secondary neurons of the ventral MDH and paratrigeminal nucleus to allow the reception of afferent information from these other nasal nerves. After bilateral sectioning of the AEN, brainstem areas that are part of the efferent aspect of the diving response could have been activated by a couple of possible mechanisms. After neuronal plasticity the newly reactivated secondary neurons within the ventral MDH and paratrigeminal nucleus could activate efferent brainstem nuclei in the same way they would have had they received AEN afferent information. Alternatively, there could be a descending suprabulbar component to the central neuronal circuitry that is only unmasked after the AENs are sectioned bilaterally. Clearly, future areas of research will need to investigate the central projections of the other nerves that innervate the nose and nasal passages, the potential neuronal plasticity that occurs in the secondary neurons within the ventral MDH and paratrigeminal nucleus, and the descending suprabulbar pathways that can activate brainstem neurons involved in cardiorespiratory control during diving.

## Author contributions

All authors (PM, EW, and KD) made substantial contributions to this manuscript, including data acquisition and manuscript generation and review. PM and EW conceptualized the experimental design of this project. EW and PM trained the rats to dive through the maze and conducted the diving experiments. KD conducted surgical experiments to cut the AEN. KD and EW conducted the Fos immunohistochemistry. KD and EW generated photomicrographic data. EW and PM analyzed the photomicrographs and counted the Fos neurons within the selected brainstem regions. EW wrote and KD reviewed initial drafts of the manuscript. PM produced the final versions of all photomicrographic figures and was responsible for generating the final version of the submitted manuscript.

### Conflict of interest statement

The authors declare that the research was conducted in the absence of any commercial or financial relationships that could be construed as a potential conflict of interest.

## Abbreviations

7: Facial nucleus
7n: Facial nerve
12: Hypoglossal nucleus
A5: A5 noradrenergic area
AEN: Anterior Ethmoidal Nerve
AP: Area Postrema
bc: brachium conjunctivum
CPA: caudal pressor area
cNTS: commissural subregion of Nucleus Tractus Solitarius
dlNTS: dorsal lateral subregion of Nucleus Tractus Solitarius
KF: Kölliker-Fuse nucleus
LC: Locus Coeruleus
LPBel: external lateral subregion of the lateral Parabrachial nucleus
LPBsl: superior lateral subregion of the lateral Parabrachial nucleus
LRt: Lateral reticular nucleus
LSO: Lateral superior olive
MDH: Medullary Dorsal Horn
mNTS: medial subregion of the Nucleus Tractus Solitarius
MPB: medial Parabrachial nucleus
NA: Nucleus ambiguus
NTS: Nucleus Tractus Solitarius
PB: Parabrachial region
Ra: expanded Raphe region
RVLM: Rostral Ventrolateral Medulla
sol: Solitary tract
sp5: spinal trigeminal tract
Sp5I: Spinal trigeminal nucleus Interpolaris
vlNTS: ventral lateral subregion of Nucleus Tractus Solitarius
vPara: paratrigeminal nucleus within ventral spinal trigeminal tract.

## References

[B1] Aicher S. Milner T. Pickel V. Reis D. (2000). Anatomical substrates for baroreflex sympathoinhibition in the rat. Brain Res. Bull. 51, 107–110. 10.1016/S0361-9230(99)00233-6 10709955

[B2] Andresen M. C. Kunze D. L. (1994). Nucleus tractus solitarius - gateway to neural circulatory control. Ann. Rev. Physiol. 56, 93–116. 10.1146/annurev.ph.56.030194.000521 7912060

[B3] Andresen M. Doyle M. Bailey T. Jin Y.-H. (2004). Differentiation of autonomic reflex control begins with cellular mechanisms at the first synapse within the nucleus tractus solitarius. Br. J. Med. Biol. Res. 37, 549–558. 10.1590/S0100-879X2004000400012 15064818

[B4] Anton F. Peppel P. (1991). Central projections of trigeminal primary afferents innervating the nasal mucosa: a horseradish peroxidase study in the rat. Neuroscience 41, 617–628. 10.1016/0306-4522(91)90354-Q 1714553

[B5] Aston-Jones G. Shipley M. T. Grzanna R. (1995). The locus coeruleus, A5 and A7 noradrenergic cell groups, in The Rat Nervous System, 2nd Edn., ed Paxinos G. (Sydney: Academic Press), 183–213.

[B6] Butler P. J. Jones D. R. (1997). Physiology of diving birds and mammals. Physiol. Rev. 77, 837–899. 9234967 10.1152/physrev.1997.77.3.837

[B7] Chan-Palay V. (1978). The paratrigeminal nucleus. I. Neurons and synaptic organization. J. Neurocytol. 7, 405–418. 10.1007/BF01173988 99495

[B8] Chotiyanonta J. S. DiNovo K. M. McCulloch P. F. (2013). Bilateral sectioning of the anterior ethmoidal nerves does not eliminate the diving response in voluntarily diving rats. Physiol. Rep. 1:e00141 10.1002/phy2.141 24400143 PMC3871456

[B9] Ciriello J. Hochstenbach S. L. Roder S. (1994). Central projections of baroreceptor and chemoreceptor afferent fibers in the rat, in Nucleus of the Solitary Tract, ed Barraco I. R. A. (Boca Raton, FL: CRC Press), 35–50.

[B10] De Sousa Buck H. Caous C. A. Lindsey C. J. (2001). Projections of the paratrigeminal nucleus to the ambiguus, rostroventrolateral and lateral reticular nuclei, and the solitary tract. Auton. Neurosci. 87, 187–200. 10.1016/S1566-0702(00)00259-9 11476279

[B11] Dragunow M. Faull R. (1989). The use of c-fos as a metabolic marker in neuronal pathway tracing. J. Neurosci. Methods 29, 261–265. 10.1016/0165-0270(89)90150-7 2507830

[B12] Dutschmann M. Herbert H. (1996). The Kölliker-Fuse nucleus mediates the trigeminally induced apnoea in the rat. Neuroreport 7, 1432–1436. 10.1097/00001756-199605310-00022 8856692

[B13] Dutschmann M. Herbert H. (1997). Fos expression in the rat parabrachial and Kölliker-Fuse nuclei after electrical stimulation of the trigeminal ethmoidal nerve and water stimulation of the nasal mucosa. Exp. Brain Res. 117, 97–110. 10.1007/s002210050203 9386008

[B14] Dutschmann M. Herbert H. (1998). The medial nucleus of the solitary tract mediates the trigeminally evoked pressor response. Neuroreport 9, 1053–1057. 9601666

[B15] Dutschmann M. Herbert H. (2006). The Kölliker-Fuse nucleus gates the postinspiratory phase of the respiratory cycle to control inspiratory off-switch and upper airway resistance in rat. Eur. J. Neurosci. 24, 1071–1084. 10.1111/j.1460-9568.2006.04981.x 16930433

[B16] Dutschmann M. Paton J. F. (2002). Influence of nasotrigeminal afferents on medullary respiratory neurones and upper airway patency in the rat. Pflugers Arch. Eur. J. Physiol. 444, 227–235. 10.1007/s00424-002-0797-x 11976936

[B17] Furilla R. A. Jones D. R. (1986). The contribution of nasal receptors to the cardiac response to diving in restrained and unrestrained redhead ducks (Aythya americana). J. Exp. Biol. 121, 227–238. 3958677 10.1242/jeb.121.1.227

[B18] Gordon F. J. McCann L. A. (1988). Pressor responses evoked by microinjections of L-glutamate into the caudal ventrolateral medulla of the rat. Brain Res. 457, 251–258. 10.1016/0006-8993(88)90693-2 2905917

[B19] Gorini C. Jameson H. S. Mendelowitz D. (2009). Serotonergic modulation of the trigeminocardiac reflex neurotransmission to cardiac vagal neurons in the nucleus ambiguus. J. Neurophysiol. 102, 1443–1450. 10.1152/jn.00287.2009 19553488 PMC2746775

[B20] Greene E. C. (1963). Anatomy of the Rat. New York, NY: Hafner.

[B21] Guyenet P. G. (2006). The sympathetic control of blood pressure. Nat. Rev. Neurosci. 7, 335–346. 10.1038/nrn1902 16760914

[B22] Guyenet P. G. (2014). Regulation of breathing and autonomic outflows by chemoreceptors. Compr. Physiol. 4, 1511–1562. 10.1002/cphy.c140004 25428853 PMC4794276

[B23] Guyenet P. G. Stornetta R. L. Bochorishvili G. Depuy S. D. Burke P. G. Abbott S. B. (2013). C1 neurons: the body's EMTs. Am. J. Physiol. Regul. Integr. Comp. Physiol. 305, R187–R204. 10.1152/ajpregu.00054.2013 23697799 PMC3743001

[B24] Hilaire G. Viemari J. C. Coulon P. Simonneau M. Bevengut M. (2004). Modulation of the respiratory rhythm generator by the pontine noradrenergic A5 and A6 groups in rodents. Resp. Physiol. Neurobiol. 143, 187–197. 10.1016/j.resp.2004.04.016 15519555

[B25] Hollandsworth M. P. DiNovo K. M. McCulloch P. F. (2009). Unmyelinated fibers of the anterior ethmoidal nerve in the rat co-localize with neurons in the medullary dorsal horn and ventrolateral medulla activated by nasal stimulation. Brain Res. 1298, 131–144. 10.1016/j.brainres.2009.08.077 19732757 PMC2760627

[B26] Itoi K. Sugimoto N. (2010). The brainstem noradrenergic systems in stress, anxiety and depression. J. Neuroendocrinol. 22, 355–361. 10.1111/j.1365-2826.2010.01988.x 20210846

[B27] Iwata K. Miyachi S. Imanishi M. Tsuboi Y. Kitagawa J. Teramoto K.. (2011). Ascending multisynaptic pathways from the trigeminal ganglion to the anterior cingulate cortex. Exp. Neurol. 227, 69–78. 10.1016/j.expneurol.2010.09.013 20854814

[B28] Junior A. B. Caous C. A. Yu Y.-G. Lindsey C. J. (2004). Barosensitive neurons in the rat tractus solitarius and paratrigeminal nucleus: a new model for medullary, cardiovascular reflex regulation. Can. J. Physiol. Pharmacol. 82, 474–484. 10.1139/y04-054 15389294

[B29] Kanbar R. Depuy S. D. West G. H. Stornetta R. L. Guyenet P. G. (2011). Regulation of visceral sympathetic tone by A5 noradrenergic neurons in rodents. J. Physiol. 589, 903–917. 10.1113/jphysiol.2010.198374 21173073 PMC3060369

[B30] Kovacs K. (2008). Measurement of immediate-early gene activation- c-fos and beyond. J. Neuroendocrinol. 20, 665–672. 10.1111/j.1365-2826.2008.01734.x 18601687

[B31] Loewy A. D. Mckellar S. Saper C. B. (1979). Direct projections from the A5 catecholamine cell group to the intermediolateral cell column. Brain Res. 174, 309–314. 10.1016/0006-8993(79)90852-7 487131

[B32] McCulloch P. F. (2005). Activation of the trigeminal medullary dorsal horn during voluntary diving in rats. Brain Res. 1051, 194–198. 10.1016/j.brainres.2005.05.059 15978555

[B33] McCulloch P. F. (2012). Animal models for investigating the central control of the mammalian diving response. Front. Physiol. 3:169. 10.3389/fphys.2012.00169 22661956 PMC3362090

[B34] McCulloch P. F. (2014). Training rats to voluntarily dive underwater: investigations of the mammalian diving response. J. Vis. Exp. e52093. 10.3791/52093 25407626 PMC4354053

[B35] McCulloch P. F. DiNovo K. M. Connolly T. M. (2010). The cardiovascular and endocrine responses to voluntary and forced diving in trained and untrained rats. Am. J. Physiol. Reg. Integr. Comp. Physiol. 298, R224–R234. 10.1152/ajpregu.00592.2009 19923359 PMC2806205

[B36] McCulloch P. F. DiNovo K. M. Westerhaus D. J. Vizinas T. A. Peevey J. F. Lach M. A.. (2013). Trigeminal Medullary Dorsal Horn Neurons Activated by Nasal Stimulation Coexpress AMPA, NMDA, and NK1 Receptors. ISRN Neurosci. 2013, e52093 10.1155/2013/152567 24967301 PMC4045565

[B37] McCulloch P. F. Jones D. R. (1990). Cortical influences on diving bradycardia in muskrats (Ondatra zibethicus). Physiol. Zool. 63, 1098–1117. 10.1086/physzool.63.6.30152635

[B38] McCulloch P. F. Panneton W. M. (1997). FOS immunohistochemical determination of brainstem neuronal activation in the muskrat after nasal stimulation. Neuroscience 78, 913–925. 10.1016/S0306-4522(96)00633-1 9153669

[B39] McCulloch P. F. Panneton W. M. (2003). Activation of brainstem catecholaminergic neurons during voluntary diving in rats. Brain Res. 984, 42–53. 10.1016/S0006-8993(03)03051-8 12932838

[B40] McCulloch P. F. Panneton W. M. Guyenet P. G. (1999). The rostral ventrolateral medulla mediates the sympathoexcitation produced by chemical stimulation of the rat nasal mucosa. J. Physiol. 516, 471–484. 10.1111/j.1469-7793.1999.0471v.x 10087346 PMC2269263

[B41] Natarajan M. Morrison S. F. (2000). Sympathoexcitatory CVLM neurons mediate responses to caudal pressor area stimulation. Am. J. Physiol. Regul. Integr. Comp. Physiol. 279, R364–R374. 10938222 10.1152/ajpregu.2000.279.2.R364

[B42] Nattie E. Li A. (2006). Central chemoreception 2005: a brief review. Auton. Neurosci. 126, 332–338. 10.1016/j.autneu.2006.02.003 16581308

[B43] Nattie E. Li A. (2012). Central chemoreceptors: locations and functions. Compr. Physiol. 2, 221–254. 10.1002/cphy.c100083 23728974 PMC4802370

[B44] Panneton W. M. (1991). Primary afferent projections from the upper respiratory tract in the muskrat. J. Comp. Neurol. 308, 51–65. 10.1002/cne.903080106 1714922

[B45] Panneton W. M. (2013). The mammalian diving response: an enigmatic reflex to preserve life? Physiology 28, 284–297. 10.1152/physiol.00020.2013 23997188 PMC3768097

[B46] Panneton W. M. Anch A. M. Panneton W. M. Gan Q. (2014). Parasympathetic preganglionic cardiac motoneurons labeled after voluntary diving. Front. Physiol. 5:8. 10.3389/fphys.2014.00008 24478721 PMC3904087

[B47] Panneton W. M. Gan Q. (2014). Direct reticular projections of trigeminal sensory fibers immunoreactive to CGRP: potential monosynaptic somatoautonomic projections. Front. Neurosci. 8:136. 10.3389/fnins.2014.00136 24926231 PMC4046267

[B48] Panneton W. M. Gan Q. Dahms T. E. (2010a). Cardiorespiratory and neural consequences of rats brought past their aerobic dive limit. J. Appl. Physiol. 109, 1256–1269. 10.1152/japplphysiol.00110.2010 20705947 PMC2971699

[B49] Panneton W. M. Gan Q. Juric R. (2006). Brainstem projections from recipient zones of the anterior ethmoidal nerve in the medullary dorsal horn. Neuroscience 141, 889–906. 10.1016/j.neuroscience.2006.04.055 16753263

[B50] Panneton W. M. Gan Q. Juric R. (2010b). The rat: a laboratory model for studies of the diving response. J. Appl. Physiol. 108, 811–820. 10.1152/japplphysiol.00600.2009 20093670 PMC2853196

[B51] Panneton W. M. Gan Q. Le J. Livergood R. S. Clerc P. Juric R. (2012a). Activation of brainstem neurons by underwater diving in the rat. Front. Physiol. 3:111. 10.3389/fphys.2012.00111 22563319 PMC3342523

[B52] Panneton W. M. Gan Q. Sun D. W. (2012b). Persistence of the nasotrigeminal reflex after pontomedullary transection. Respir. Physiol. Neurobiol. 180, 230–236. 10.1016/j.resp.2011.11.012 22154693 PMC3273655

[B53] Panneton W. M. McCulloch P. F. Sun W. (2000). Trigemino-autonomic connections in the muskrat: the neural substrate for the diving response. Brain Res. 874, 48–65. 10.1016/S0006-8993(00)02549-X 10936223

[B54] Panneton W. M. Sun W. Gan Q. (2008). Pressor responses to nasal stimulation are unaltered after disrupting the CPA. Auton. Neurosci. 144, 13–21. 10.1016/j.autneu.2008.08.002 18809361 PMC2678971

[B55] Paxinos G. Watson C. (1998). The Rat Brain in Stereotaxic Coordinates. New York, NY: Academic Press.

[B56] Phelan K. D. Falls W. M. (1989). The interstitial system of the spinal trigeminal tract in the rat: anatomical evidence for morphological and functional heterogeneity. Somatosens. Mot. Res. 6, 367–399. 10.3109/08990228909144682 2547273

[B57] Pilowsky P. M. (2014). Peptides, serotonin, and breathing: the role of the raphe in the control of respiration. Cen. Nerv. Syst. Control Resp. 209, 169. 10.1016/B978-0-444-63274-6.00009-6 24746048

[B58] Potts J. T. (2001). Exercise and sensory integration. Ann. N.Y. Acad. Sci. 940, 221–236. 10.1111/j.1749-6632.2001.tb03679.x 11458680

[B59] Potts J. T. (2002). Neural circuits controlling cardiorespiratory responses: baroreceptor and somatic afferents in the nucleus tractus solitarius. Clin. Exp. Pharmacol. Physiol. 29, 103–111. 10.1046/j.1440-1681.2002.03613.x 11906467

[B60] Richerson G. B. (2004). Serotonergic neurons as carbon dioxide sensors that maintain pH homeostasis. Nat. Rev. Neurosci. 5, 449–461. 10.1038/nrn1409 15152195

[B61] Richerson G. B. Wang W. Hodges M. R. Dohle C. I. Diez-Sampedro A. (2005). Homing in on the specific phenotype(s) of central respiratory chemoreceptors. Exp. Physiol. 90, 259–566. 10.1113/expphysiol.2005.029843 15728134

[B62] Rinaman L. (2011). Hindbrain noradrenergic A2 neurons: diverse roles in autonomic, endocrine, cognitive, and behavioral functions. Am. J. Physiol. Regul. Integr. Comp. Physiol. 300, R222–R235. 10.1152/ajpregu.00556.2010 20962208 PMC3043801

[B63] Rybka E. J. McCulloch P. F. (2006). The anterior ethmoidal nerve is necessary for the initiation of the nasopharyngeal response in the rat. Brain Res. 1075, 122–132. 10.1016/j.brainres.2005.12.112 16466647

[B64] Saxon D. W. Hopkins D. A. (2006). Ultrastructure and synaptology of the paratrigeminal nucleus in the rat: primary pharyngeal and laryngeal afferent projections. Synapse 59, 220–234. 10.1002/syn.20233 16385507

[B65] Shigenaga Y. Okamoto T. Nishimori T. Suemune S. Nasution I. D. Chen I. C.. (1986). Oral and facial representation in the trigeminal principal and rostral spinal nuclei of the cat. J. Comp. Neurol. 244, 1–18. 10.1002/cne.902440102 3950088

[B66] Shoemaker J. K. Goswami R. (2015). Forebrain neurocircuitry associated with human reflex cardiovascular control. Front. Physiol. 6:240. 10.3389/fphys.2015.00240 26388780 PMC4555962

[B67] Smith J. C. Abdala A. P. Borgmann A. Rybak I. A. Paton J. F. (2013). Brainstem respiratory networks: building blocks and microcircuits. Trends Neurosci. 36, 152–162. 10.1016/j.tins.2012.11.004 23254296 PMC4080795

[B68] Sun M. K. (1995). Central neural organization and control of sympathetic nervous system in mammals. Progr. Neurobiol. 47, 157–233. 10.1016/0301-0082(95)00026-8 8719915

[B69] Sun W. Panneton W. M. (2002). The caudal pressor area of the rat: its precise location and projections to the ventrolateral medulla. Am. J. Physiol. Regul. Integr. Comp. Physiol. 283, R768–R778. 10.1152/ajpregu.00184.2002 12185012

[B70] Verberne A. J. M. Owens N. C. (1998). Cortical modulation of the cardiovascular system. Prog. Neurobiol. 54, 149–168. 10.1016/S0301-0082(97)00056-7 9481796

[B71] Wasserman A. M. Sahibzada N. Hernandez Y. M. Gillis R. A. (2000). Specific subnuclei of the nucleus tractus solitarius play a role in determining the duration of inspiration in the rat. Brain Res. 880, 118–130. 10.1016/S0006-8993(00)02782-7 11032996

[B72] Yu Y. G. Lindsey C. J. (2003). Baroreceptor-sensitive neurons in the rat paratrigeminal nucleus. Auton. Neurosci. 105, 25–34. 10.1016/S1566-0702(03)00022-5 12742188

